# Involution of retinopathy of prematurity and neurodevelopmental outcomes after intravitreal bevacizumab treatment

**DOI:** 10.1371/journal.pone.0223972

**Published:** 2019-10-16

**Authors:** Yu-Shan Chang, Ying-Tin Chen, Tso-Ting Lai, Hung-Chieh Chou, Chien-Yi Chen, Wu-Shiun Hsieh, Chung-May Yang, Po-Ting Yeh, Po-Nien Tsao

**Affiliations:** 1 Department of Pediatrics, National Cheng Kung University Hospital, Tainan, Taiwan; 2 Department of Pediatrics, China Medical University Children's Hospital, Taichung, Taiwan; 3 Department of Ophthalmology, National Taiwan University Hospital, Taipei, Taiwan; 4 Department of Pediatrics, National Taiwan University Children’s Hospital, Taipei, Taiwan; 5 The Research Center for Developmental Biology and Regenerative Medicine, National Taiwan University, Taipei, Taiwan; Children's Hospital Boston, UNITED STATES

## Abstract

This single-centered, retrospective cohort study investigated the timing of involution of retinopathy of prematurity (ROP) and retinal vascularization to zone III after intravitreal bevacizumab (IVB) treatment and its possible impacts on postnatal growth and neurodevelopment. Premature infants with birth weight ≤1500 g, born between 2008 to 2014 and diagnosed with ROP were enrolled. All patients with type 1 ROP underwent IVB as 1^st^ line treatment and were recruited as the study group; those with any stage of ROP except type 1 ROP without treatment served as controls. Neurodevelopmental outcomes were assessed using the Bayley Score of Infant Development (BSID) editions II or III. The study group included 35 eyes from 18 patients; the control group included 86 patients. Twenty-three eyes (65.7%) exhibited ROP regression after a single dose of IVB. The majority of plus sign and extraretinal neovascularization regressed within two weeks. The length of time for retinal vascularization to reach zone III was significantly longer in the treatment group compared with the control (mean post-menstruation age 54.5 vs. 47.0 weeks, p<0.001). Long-term follow-up showed no significant differences in body weight and neurodevelopment between the study and control groups up to the 2-year corrected age.

## Introduction

Retinopathy of prematurity (ROP) remains a leading cause of childhood blindness. Most cases of ROP occur in extremely low-gestational-age neonates (gestational age of less than 28 weeks at birth) [1–5]. Laser photocoagulation is currently the standard of treatment for ROP. Nowadays, the widely-accepted treatment criteria is designated as type 1 ROP, as established in the ETROP study [6, 7]. In 2007, anti–vascular endothelial growth factor (VEGF) intravitreal therapy emerged as a new treatment modality for ROP [8]. The BEATROP (Bevacizumab Eliminates the Angiogenic Threat of Retinopathy of Prematurity) trial showed significant benefits of intravitreal bevacizumab (IVB) monotherapy for Stage 3+, Zone I disease [9]. The advantages of VEGF inhibitors for ROP treatment, compared with laser treatment, include less time to administer treatment, less treatment-related destruction of the peripheral retina, and a lower likelihood of high myopia and astigmatism [10]. However, there are concerns about the long-term effects on ocular health, long-term visual outcome, systemic safety, and potential influence on the neurodevelopment of VEGF inhibitors, especially when given so early in life.

The aim of our study was to report the timing of involution of ROP and achievement of full retinal vascularization after IVB treatment, as well as its possible influence on postnatal growth and neurodevelopmental outcomes.

## Patients and methods

### Study population

This was a single-centered, retrospective cohort study. We collected inborn, premature infants with birth weight ≤1500 g, born between 2008 to 2014, who had been diagnosed with any stage of ROP from 2008 to 2014 at National Taiwan University Children’s Hospital. Patients with major congenital anomalies, cyanotic heart disease, death before discharge, history of suspected congenital infection, those who died before they reached 1 year of age (unable to complete neurodevelopmental assessment), and those receiving other investigational treatment for ROP except IVB were excluded. Our ROP screening criteria were: (1) Gestational age ≤ 32 weeks or birth weight ≤ 1500g (2) Gestational age ≤ 35 weeks or birth weight ≤ 2000g who had received oxygen supplementation during hospitalization. The indications for treatment were patients whose retinopathy met the criteria (type 1 ROP) established by the Early Treatment for Retinopathy of Prematurity Study (ETROP).[6, 7] Treatment was initiated within 72 hours of detection [11]. All patients were initially examined by retina fellows from the department of ophthalmology. However, the staging, treatment procedure, additional treatment determination, and the final vascularization status were performed by a single experienced retina specialist (PTY). All patients with type 1 ROP received intravitreal bevacizumab (IVB) as the first-line treatment and were enrolled as the study group. Those with any stage of ROP except type 1 ROP who did not receive any form of treatment were enrolled as controls.

### Procedure—Intravitreal bevacizumab treatment

Eyes were prepared in a standard fashion using 5% povidone–iodine and topical antibiotics, after which 0.625 mg (0.025 mL) bevacizumab was injected intravitreally via the pars plicata under intravenous sedation. The injection was performed with a 30-gauge needle that was initially directed along an angle perpendicular to the globe 1.5 mm behind the limbus and then redirected slightly toward the optic nerve after the needle had entered the sclera. This technique was used to avoid damaging both the lens and retina [12].

### Evaluation and management after intravitreal bevacizumab treatment

After treatment, patients were monitored every 3–7 days followed by follow-up every 1 or 2 weeks until vascularization into zone III was observed [13]. ROP involution was defined as regression in disease severity by at least one stage and absence of plus disease.[13] ROP progression was defined as vascularization with the presence of a new demarcation line, ridge, or extraretinal fibrovascular proliferation with or without plus disease [13].

If the patients did not respond positively to the IVB injection, which was defined as plus disease or fibrovascular proliferation persisting for 2 weeks after primary treatment, or if the ROP worsened, the patients were re-treated with the conventional laser treatment, an additional IVB injection, scleral buckling, or a lens-sparing vitrectomy, depending on the clinical condition. If the patient had recurrent or persistent type 1 ROP, he or she received either additional IVB or laser treatment depending on the decision of the patient’s parents after a thorough discussion of the pros and cons of both options. If the patient progressed to stage IV ROP, scleral buckling or lens-sparing vitrectomy was performed.

### Data collection

Clinical characteristics and risk factors for ROP, including gestational age, birth weight, neonatal morbidities, chorioamnionitis, sepsis and oxygen requirements, were collected via a retrospective chart review. Body weight was recorded during outpatient follow-ups for either routine vaccinations or neurodevelopment evaluations. The evaluation of neurodevelopment was performed with the Bayley Scales of Infant and Toddler Development, second edition (Bayley-II) for those born before 2011 and the third edition (Bayley-III) for those born after 2011. Certified pediatric psychologists and pediatricians who were experienced with this assessment performed the assessment at corrected ages of 6 months, 1 year, and 2 years. The Bayley-II was composed of two parts: a mental development index (MDI) and a psychomotor development index (PDI). The Bayley-III yielded three composite scores: cognitive, language, and motor. Neurodevelopmental impairment (NDI) was defined as the presence of any of the following: cerebral palsy, sensorineural/mixed hearing loss, any MDI or PDI <70, or any Bayley-III composite score <85.

### Statistical analyses

SPSS version 17.0 was used for the statistical analysis of the data. Infant demographic characteristics, growth, and the neurodevelopmental outcomes of the two groups were compared by using a Fisher’s exact test (categorical variables) and a Wilcoxon Rank Sum test (continuous variables) due to the relatively small sample size. Continuous variables were expressed as a median (IQR) and categorical variables as a number (%).

### Ethical considerations

This retrospective study was approved by the Institutional Review Board of National Taiwan University Children’s Hospital (201708065RINA). This research was conducted adhering to the principles expressed in the Declaration of Helsinki. Written informed consent was obtained from all parents whose children were receiving the bevacizumab treatment.

## Results

**Fig 1:**
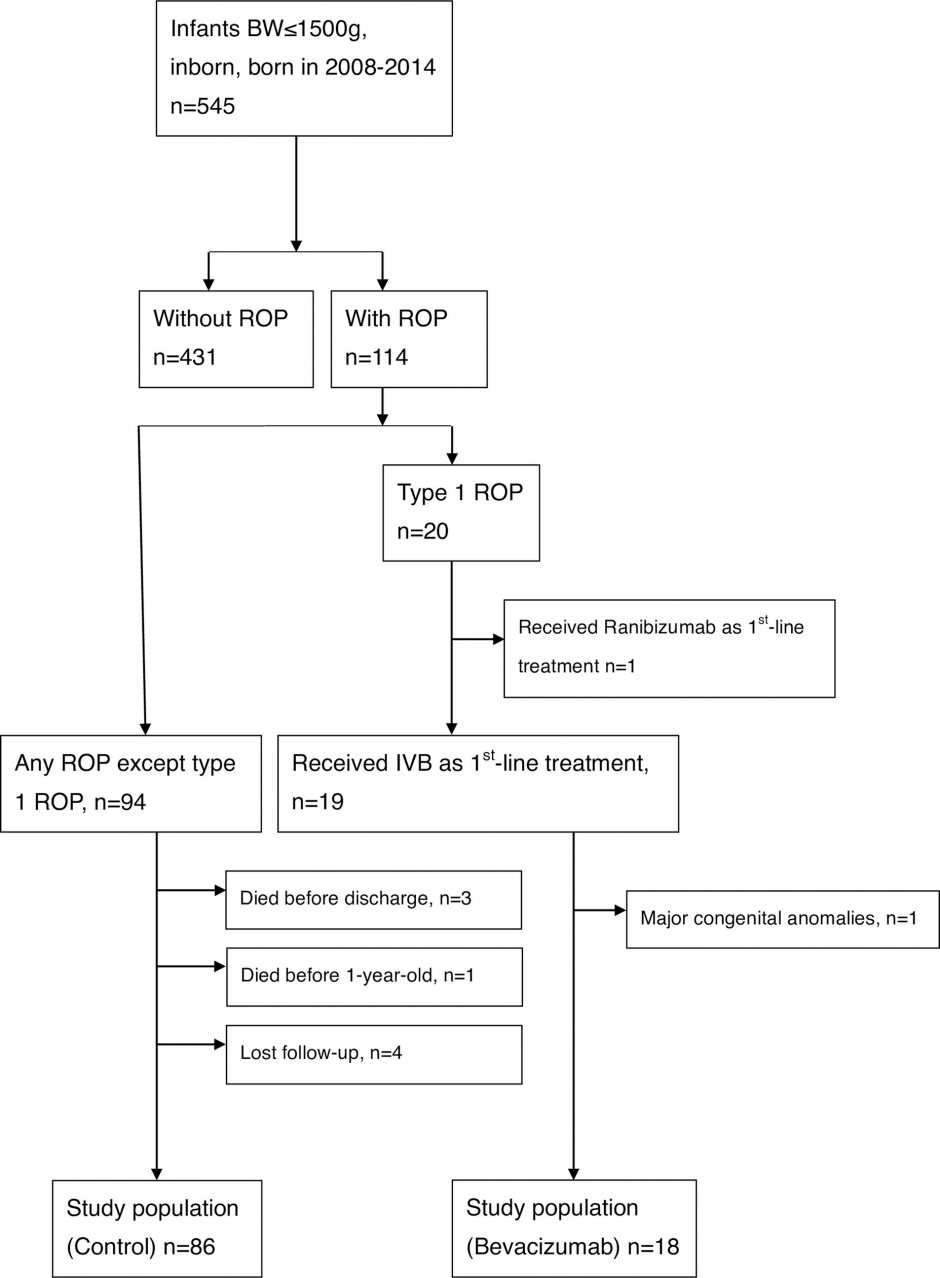
Patient enrollment algorithm. In total, 104 infants (bevacizumab, n = 18; control, n = 86) entered the final analysis conducted in this study.

During the study period, 545 inborn preterm infants with birth weights ≤1500 g were admitted to the NICU at National Taiwan University Children’s Hospital, which is a tertiary medical center in Taiwan. Overall, 114 were diagnosed with ROP. Among them, 20 were treated for type 1 ROP; 94 who were diagnosed with any stage of ROP except type 1 ROP and did not receive any treatment were recruited as controls. We excluded patients who received ranibizumab as a first-line treatment (n = 1), those with major congenital anomalies (n = 1, idiopathic liver failure), those who died before discharge (n = 3) or within 1 year (n = 1), and those with no follow-up data (n = 3). Follow-up data were available for 104 infants (bevacizumab, n = 18; control, n = 86). Overall, thirty-five eyes from eighteen patients received first-line treatment with bevacizumab. The enrollment algorithm is shown in Fig 1.

### Patient characteristics and neonatal comorbidity

Table 1 compares the neonatal characteristics of the preterm infants who were treated with bevacizumab versus the control. The bevacizumab-treated infants had significantly lower gestational age and birth weight. Among them, more were born to multiple pregnancies. They also required longer respiratory support, had higher peak oxygen demands, and stayed longer in the NICU. Rates of neonatal complications were similar with the exception of the incidence of fungemia, which was higher in the bevacizumab group.

**Table 1:** Neonatal characteristics of infants treated with bevacizumab versus the control (2008–2014).

	Bevacizumab	Control	*p*-value
**No. of patients**	18	86	-
**No. of eyes treated with IVB**	35	0	-
**Neonatal characteristics**			
GA (week) BW (g) Male gender Multiple births SGA	24.5 (24–25) 641 (595–722) 11 (61) 13 (72) 6 (33)	27.5 (25–30) 990 (761–1204) 40 (47) 34 (40) 27 (31)	<0.001 <0.001 0.307 0.018 1.000
**Maternal characteristics**			
Received prenatal steroid	17 (94)	74 (86)	0.458
Preeclampsia	4 (22)	16 (19)	0.746
Chorioamnionitis	3 (17)	6 (7)	0.186
**Maternal education**			
Less than high school	1 (6)	2 (2)	0.533
High school	7 (39)	39 (45)	
College and above	10 (56)	45 (52)	
**Morbidities**			
RDS BPD IVH (grade≥3) hsPDA^a^ LOS Fungemia NEC (stage≥2) Cystic PVL SIP	17 (94) 11 (61) 3 (17) 13 (72) 8 (44) 4 (22) 2 (11) 3 (17) 0 (0)	73 (85) 39 (45) 9 (10) 45 (52) 26 (30) 4 (5) 2 (2) 9 (10) 2 (2)	0.455 0.301 0.432 0.191 0.275 0.029 0.138 0.432 1.000
**Respiratory support**^b^			
No. of days	93.5 (76.5–127.8)	56.0 (23.5–84.8)	<0.001
Peak FiO2 requirement (%)	60.0 (42.5–77.5)	30.0 (21.3–70.0)	0.005
NICU days (d)	96.0 (84.8–134.3)	63 (41.3–89.8)	<0.001

Continuous variables were expressed as a median (IQR) and categorical variables were expressed as a number (%). GA, gestational age; BW, birth weight; SGA, small for gestational age; RDS, respiratory distress syndrome; BPD, bronchopulmonary dysplasia; IVH, intraventricular hemorrhage; LOS, late-onset sepsis; NEC, necrotizing enterocolitis; PVL, periventricular leukomalacia; SIP, spontaneous intestinal perforation; NICU, neonatal intensive care unit

hsPDA^a^, hemodynamically significant patent ductus arteriosus requiring medical or surgical treatment

Respiratory support^b^, O2 supplement, non-invasive ventilation (nasal continuous positive airway pressure, nasal intermittent positive airway pressure, bi-level positive airway pressure), mechanical ventilation

Next, we compared the neonatal characteristics of only infants born after 2011 (all of which received the Bayley-III assessment). Bevacizumab-treated infants still had significantly lower gestational age and birth weight and required longer respiratory support and NICU stays. There were no significant between-group differences in neonatal morbidities (table in S1 Table).

### Structural outcomes

Among the treated eyes, all eyes (100%) were stage III+, zone II ROP. The median age of treatment was 38 (34–65) weeks post-menstruation age (PMA).

**Fig 2:**
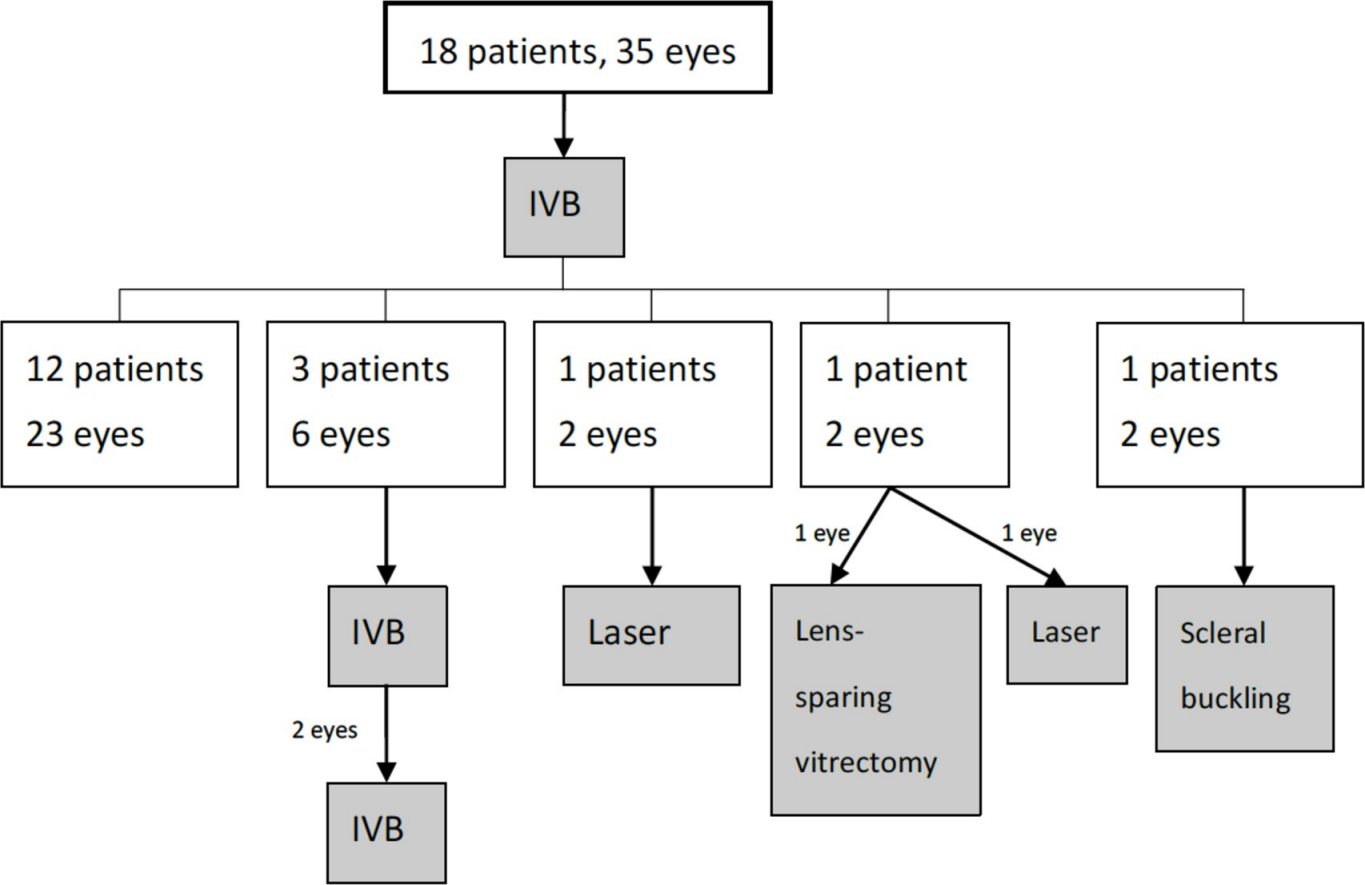
Algorithm demonstrating the structural outcomes after intravitreal bevacizumab treatment.

Twenty-three eyes (65.7%) exhibited ROP regression after a single dose of IVB. Repeated IVB injections were required in 6 eyes (17.1%). Additional laser treatment was required in 3 eyes (8.6%), in which there was either no positive response to IVB injection or a worsening of the ROP observed. The additional laser treatments were given at PMA 45 wks, 46 wks, and 46 wks, respectively. The latter two eyes belonged to the same patient. The interval between IVB and laser treatment was 69, 41, and 41 days. Three eyes (8.8%) in two patients progressed to stage 4 ROP and required vitrectomy or scleral buckling to reattach the retinas. The time to surgery was 45, 69, and 69 weeks PMA in these 3 eyes. The latter two eyes belonged to the same patient. The interval between IVB and surgery was 69, 23, and 23 days. Fig 2 shows the algorithm demonstrating the structural outcomes of these patients.

The median timing for regression of plus sign and extraretinal fibrovascular proliferation after IVB was 4 (1–23) days and 14 (9–34) days, respectively.

Major ocular complications that were associated with IVB injection including vitreous hemorrhage occurred in 2 eyes (5.4%).

The length of time for retinal vascularization to reach zone III was significantly longer in the treatment group compared with the control (*p*<0.001). The mean PMA for retinal vascularization to zone III was 54.5 (46–85) weeks and 47.0 (38–94) weeks in the treatment group and control group, respectively.

### Long-term effects on growth and neurodevelopmental outcomes

The Bayley Scales of Infant and Toddler Development, second edition (Bayley-II) was used for those born before 2011, and the third edition (Bayley-III) was used for those born after 2011. Among the Bevacizumab-treated group, 5 patients received Bayley-II, and 13 received the Bayley-III assessment. Among the control group, 35 received Bayley-II and 51 received the Bayley-III assessment.

There were no significant differences in the Bayley-III cognitive, language, and motor composite scores between bevacizumab-treated infants and controls (Table 2). Similarly, no significant differences were found in the Bayley-II MDI and PDI scores with the exception of a lower PDI at the 1-year corrected age in the treatment group (table in S2 Table). However, the incidence of any Bayley-II PDI < 70 was significantly higher in the Bevacizumab group (Table 3).

**Table 2:** Comparison of Bayley-III composite scores.

	Bevacizumab	Control	*p*-value
	(n = 13)	(n = 51)	
**Corrected age 6-month**	(n = 12)	(n = 50)	
Cognition	95.0 (88.8–101.3)	92.5 (86.3–105.0)	1.000
Language	94.0 (91.5–100.0)	97.0 (94.0–105.3)	0.194
Motor	89.5 (82.0–91.3)	88.0 (79.0–99.3)	0.682
**Corrected age 1-year**	(n = 13)	(n = 51)	
Cognition	100.0 (95.0–110.0)	100.0 (92.5–105.0)	0.928
Language	94.0 (77.0–97.0)	91.0 (86.0–98.5)	0.818
Motor	85.0 (82.0–103.0)	91.0 (85.0–94.0)	0.803
**Corrected age 2-year**	(n = 13)	(n = 51)	
Cognition	105.0 (85.0–105.0)	100.0 (90.0–110.0)	0.529
Language	86.0 (77.0–109.0)	94.0 (79.0–109.0)	0.562
Motor	85.0 (76.0–100.0)	91.0 (86.5–100.0)	0.298

Continuous variables were expressed as median (IQR).

**Table 3:** Comparison of body weight and neurodevelopmental outcomes.

	Bevacizumab (n = 18)	Control (n = 86)	*p*-value
**Body weight:**			
CA 6-month (kg)	6.75 (5.63–7.36)	7.00 (6.30–8.00)	0.190
CA 1-year (kg)	8.33 (7.25–8.83)	8.79 (7.90–9.53)^b^	0.234
CA 2-year (kg)	11.00 (9.39–11.90)^a^	10.90 (9.81–11.90)^c^	0.749
**Bayley-III <85,** n (%)			
Cognition	2/13 (15.4)	11/51 (21.6)	1.000
Language	6/13 (46.2)	19/51 (37.3)	0.751
Motor	7/13 (53.8)	24/51 (47.1)	0.761
**Bayley-II <70,** n (%)			
MDI	1/5 (20.0)	7/35 (20.0)	1.000
PDI	4/5 (80.0)	9/35 (25.7)	0.031
**Neurodevelopmental** **Impairment,** n (%)^d^	11/18 (61.1)	38/86 (44.2)	0.207

Continuous variables were expressed as a median (IQR), and categorical variables were expressed as a number (%). CA, corrected age. MDI, Mental Development Index. PDI, Psychomotor Development Index.

^a^ data available for 17 patients

^b^ data available for 84 patients

^c^ data available for 78 patients

^d^ Neurodevelopmental impairment was defined as the presence of any of the following: cerebral palsy, sensorineural/mixed hearing loss, any Bayley-II MDI or PDI <70, any Bayley-III composite score <85.

When we pooled all the patients together and compared the incidence of neurodevelopmental impairment (defined as the presence of any of the following: cerebral palsy, sensorineural/mixed hearing loss, any Bayley-II MDI or PDI <70, or any Bayley-III composite score <85), we found there was a trend toward a higher incidence of neurodevelopmental impairment in the bevacizumab-treated group, but this finding was not statistically significant. Body weights at corrected ages of 6-months, 1-year and 2-years of age were not significantly different between the bevacizumab and control groups (Table 3). We then excluded patients with IVH ≥ grade III, cystic PVL, or hydrocephalus requiring treatment in both groups and repeated the analysis. There remained no significant differences in incidence of NDI between the bevacizumab-treated and the control group (treatment group: 8/15, control: 29/76, *p*:0.389).

## Discussion

This retrospective study evaluated the involution pattern of ROP after IVB and its long-term effects on body weight growth and neurodevelopmental outcomes in preterm infants. We observed that 65.7% of the eyes under investigation showed ROP resolution after a single injection. IVB led to resolution of plus sign and extraretinal fibrovascular proliferation within two weeks. However, the treated eyes required a significantly longer time to reach zone III retinal vascularization. Body weight growth and neurodevelopmental outcomes were similar between the bevacizumab-treated group and the control up to the 2-year corrected age.

As for ocular outcomes, our study showed that IVB effectively inhibited neovascularization with prompt regression after a single injection in 65.7% of the patients. Among those that required re-treatment, the ocular outcomes were favorable with an attached retina in up to 97.1% of the eyes. A significantly longer time was needed for the eyes to achieve complete vascularization of the retina in the treatment group compared with the control group [54.5 (46–85) vs. 47.0 (38–94) weeks, p<0.001]. In a pharmacokinetics study, Miyake et al. showed in a macaque model that intravitreal injection of bevacizumab decreased the VEGF concentration in the treated eyes for at least 4 weeks [14]. Bakri et al. showed in a rabbit model that concentrations of >10μg/ml bevacizumab were maintained in the vitreous humor for 30 days after IVB [15]. Intravitreal VEGF levels dropped sharply after IVB and gradually increased to a more physiologic level to allow continued vascularization to the periphery of the retina. These studies may explain the delayed vascularization of injected eyes. Of course, we cannot exclude the possibility that severe ROP itself may delay the process of complete vascularization of the retina.

Systemic safety remains a concern for the use of anti-VEGF in preterm infants. VEGF is a key molecule involved in development of organs in infants [16]. Angiogenesis processes are also associated with neurogenesis processes [17]. Angiogenesis also appears to play a critical role in intra- and extra-uterine growth [18]. There remain debates about the potential influence of intravitreal anti-VEGF on the neurodevelopment of preterm infants. Published papers on this topic show inconsistent results [19–23]. Morin et al. compared the neurodevelopmental outcomes of patients receiving bevacizumab versus laser treatment. The authors concluded that the odds of having a severe neurodevelopmental disability were 3.1 times higher in the bevacizumab group [19]. Lien et al., however, reported no difference in neurodevelopmental outcomes between bevacizumab and laser treatment [20]. Kennedy et al., in a 2-year follow-up of a study site in the BEAT-ROP study, also found no significant difference in growth and neurodevelopment between bevacizumab and laser treatment [23]. Araz-Ersan et al. compared a laser-treatment group to a laser-with-adjuvant-bevacizumab group and found no significant difference in the Bayley-III scores in children at 2 years of age [21].

In our study, we found no significant differences between the treatment and control groups regarding either body weight or Bayley-III scores at corrected ages of 6 months, 1 year and 2 years of age, in spite of the fact that the bevacizumab-treated infants had significantly lower gestational age and birth weight. Our results are in agreement with those of three previous studies [20, 21, 23]. However, we found significantly lower Bayley-II PDI scores at the 1-year corrected age in the treatment group, as well as significantly higher incidence of any Bayley-II PDI < 70 in the treatment group. It is worth noting that 3 out of 18 patients in the treatment group required additional laser coagulation, vitrectomy, or scleral buckling after IVB due to a lack of treatment response or disease progression. These relatively severe patients were included in the comparison of body weight and neurodevelopmental outcomes. We anticipated that the potential direction of bias after including these patients into the final analysis was obtaining poorer weight gain and developmental outcomes in the treatment group. Nevertheless, we decided to include them in order to avoid underestimating the potential harm of IVB.

Several studies have shown that intravitreal bevacizumab injections suppress plasma VEGF concentrations significantly for at least 8 weeks and for as much as 12 weeks [24–28]. Interestingly, a recent study showed that VEGF serum concentrations were significantly higher upon initial detection of ROP in infants who were later treated for ROP compared to infants without ROP [29]. We may speculate that the suppression of plasma VEGF by intravitreal bevacizumab merely brings it down to the “normal” range. Wu et al. studied 8 patients with ROP and demonstrated that the baseline plasma VEGF level was close to 400 pg/ml and dropped to <100pg/ml after bevacizumab treatment [26]. Although the physiological or safety range of VEGF for premature babies is unknown, this abrupt and prolonged suppression does raise concerns. Nevertheless, further studies to determine the optimal dose of bevacizumab that is effective and has the least systemic toxicity are needed. Wallace et al. showed that a dose of bevacizumab as low as 0.031mg may be effective [30]. However, the author also reported that more patients receiving low-dose bevacizumab required additional treatment [31]. In addition, different anti-VEGF agents, for example ranibizumab and aflibercept, have demonstrated efficacy in treatment of type 1 ROP [27, 32]. Serum VEGF level was reported to be less affected by intravitreal ranibizumab and aflibercept than bevacizumab [27, 28]. However, there remain largely unsolved questions regarding risk of late recurrences and long-term safety.

In light of the BEAT-ROP study and the prevalent off-label use of IVB for treatment of ROP, our study offers important information about the long-term outcomes of bevacizumab treatment in preterm neonates. Limitations of our study include its retrospective design, small number of patients, lack of serum VEGF data, and lack of more sophisticated tools to survey the retina for patterns of vascularization. In addition, we were unable to avoid the confounding effect of severity of ROP independently on neurodevelopmental outcomes. Patients in our treatment group had lower gestational age at birth, lower birth weight (Table 1) and more severe ROP. Under the premise, we did not find significantly worse neurodevelopmental outcomes in the treatment group. We may infer from our results that at least IVB did not contribute largely to adverse neurological outcomes. The strength and value of our study include the following: To begin with, we chose those with any ROP except type I ROP as controls, instead of a laser group as in previous studies. Therefore, we were able to look solely at the effect of intravitreal bevacizumab and minimized any selection bias. Next, although retrospective and small in sample size, previous published studies were also small (patients receiving IVB: Lien et al. [20], n = 12; Morin et al. [19], n = 27, Araz-Ersan et al. [21], n = 13; Kennedy et al [23], n = 7) and drew inconsistent conclusions. Therefore, our study was able to provide further information on this issue. In addition, many previous studies evaluated neurodevelopmental outcomes only at 18-months or 24-months corrected age [19, 21, 23]. We performed evaluation at 6-months, 1-year and 2-years. Last but not least, we presented data on body weight growth after IVB treatment, which was seldom discussed in previous studies yet provides an important hint when systemic VEGF suppression is one of the major concerns of IVB.

In conclusion, our results showed prompt resolution of ROP in 65.7% of the patients after a single injection. We did not observe significant detrimental effects on body weight growth and neurodevelopmental outcomes up to 2 years after IVB. However, we found significantly lower Bayley-II PDI scores at the 1-year corrected age in the treatment group, as well as significantly higher incidence of any Bayley-II PDI < 70 in the treatment group. Therefore, there is not enough evidence to ascertain without doubt that intravitreal antiangiogenic therapy is safe for preterm infants. Further larger, prospective, controlled, randomized trials are needed to establish its long-term safety and efficacy.

## Supporting information

S1 Table Neonatal characteristics of infants born after 2011 (received the Bayley-III assessment) treated with bevacizumab versus control. (PDF)

S2 Table Comparison of Bayley-II scores. (PDF)
