# Supplementary material for: Involution of retinopathy of prematurity and neurodevelopmental outcomes after intravitreal bevacizumab treatment
Source: PLoS One. 2019 Oct 16;14(10):e0223972. doi: 10.1371/journal.pone.0223972 (PMC6795500; doi:10.1371/journal.pone.0223972)
Supplement: S1 Table — (PDF) [file pone.0223972.s001.pdf]

S1 Table Neonatal characteristics of infants born after 2011 (received the Bayley-III assessment) treated with bevacizumab versus control

	**Bevacizumab**	**Control**	***p*-value**
**No. of patients**	13	51	-
**Neonatal characteristics**			
GA (week)	25 (24-25)	26(25-29)	0.002
BW (g)	640 (520-660)	860 (717-1103)	<0.001
Male gender	7 (53.8)	26 (51.0)	1.000
Multiple births	9 (69.2)	20 (39.2)	0.067
SGA	6 (46.2)	35 (68.6)	0.343
**Maternal characteristics**			
Received prenatal steroid	12 (92.3)	45 (88.2)	1.000
Preeclampsia	4 (30.8)	8 (15.7)	0.446
Chorioamnionitis	3 (23.1)	5 (9.8)	0.343
**Maternal education**			

Less than high school	0 (0)	1 (2.0)	
High school	5 (38.5)	21 (41.2)	1.000
College and above	8 (61.5)	29 (56.9)	
**Morbidities**			
RDS	12 (92.3)	43 (84.3)	0.672
BPD	7 (53.9)	29 (56.9)	1.000
IVH (grade≥3)	2 (15.4)	6 (11.8)	0.260
hsPDA[a]	9 (69.2)	32 (62.7)	0.755
LOS	6 (46.2)	17 (33.3)	0.519
Fungemia	3 (23.1)	3 (5.9)	0.093
NEC (stage≥2)	1 (7.7)	0 (0)	0.138
Cystic PVL	2 (15.4)	7 (13.7)	1.000
SIP	0 (0)	0 (0)	1.000
**Respiratory support**[b]			
No. of days	123 (75-128)	64 (41-89)	0.001

Peak FiO2 requirement (%)	60 (40-65)	30 (21-80)	0.066
NICU days (d)	125 (90-135)	72 (47-93)	0.002

Continuous variables were expressed as median (IQR) and categorical variables as number (%). GA, gestational age; BW, birth weight; SGA, small for gestational age; RDS, respiratory distress syndrome; BPD, bronchopulmonary dysplasia; IVH, intraventricular hemorrhage; hsPDA[a], hemodynamically significant patent ductus arteriosus requiring treatment; LOS, late-onset sepsis; NEC, necrotizing enterocolitis; PVL, periventricular leukomalacia; SIP, spontaneous intestinal perforation; Respiratory support[b], O2 supplement, non-invasive ventilation (nasal continuous possible airway pressure, non-invasive positive pressure ventilation, bi-level positive airway pressure), mechanical ventilation; NICU, neonatal intensive care unit
