# Supplementary material for: Involution of retinopathy of prematurity and neurodevelopmental outcomes after intravitreal bevacizumab treatment
Source: PLoS One. 2019 Oct 16;14(10):e0223972. doi: 10.1371/journal.pone.0223972 (PMC6795500; doi:10.1371/journal.pone.0223972)
Supplement: S2 Table — (PDF) [file pone.0223972.s002.pdf]

S2 Table Comparison of Bayley-II scores

	**Bevacizumab** (n=5)	**Control** (n=35)	***p*-value**
**CA 6-month**			
	(n=4)	(n=32)	
MDI	85.0 (80.5-88.0)	90.0 (82.0-96.0)	0.178
	(n=4)	(n=32)	
PDI	79.0 (69.5-85.8)	85.0 (73.8-91.0)	0.277
**CA 1-year**			
	(n=5)	(n=29)	
MDI	80.0 (80.0-93.0)	90.0 (77.0-94.0)	0.396
	(n=5)	(n=33)	
PDI	65.0 (52.0-65.0)	81.0 (73.0-89.0)	0.025
**CA 2-year**			
	(n=4)	(n=28)	
MDI	84.0 (73.8-88.5)	86.0 (77.5-96.5)	0.286
	(n=4)	(n=28)	
PDI	72.5 (58.3-88.8)	96.0 (77.5-100.8)	0.190

Continuous variables were expressed as median (IQR). CA, corrected age; MDI, Mental Development Index; PDI, Psychomotor Development Index.
